# On the Use of Deep Learning for Imaging-Based COVID-19 Detection Using Chest X-rays

**DOI:** 10.3390/s21175702

**Published:** 2021-08-24

**Authors:** Gabriel Iluebe Okolo, Stamos Katsigiannis, Turke Althobaiti, Naeem Ramzan

**Affiliations:** 1 School of Computing, Engineering and Physical Sciences, University of the West of Scotland, Paisley PA1 2BE, UK; Naeem.Ramzan@uws.ac.uk; 2 Department of Computer Science, Durham University, Durham DH1 3LE, UK; stamos.katsigiannis@durham.ac.uk; 3 Faculty of Science, Northern Border University, Arar 91431, Saudi Arabia; Turke.althobaiti@nbu.edu.sa

**Keywords:** COVID-19, chest X-ray, deep learning, CNN, image classification

## Abstract

The global COVID-19 pandemic that started in 2019 and created major disruptions around the world demonstrated the imperative need for quick, inexpensive, accessible and reliable diagnostic methods that would allow the detection of infected individuals with minimal resources. Radiography, and more specifically, chest radiography, is a relatively inexpensive medical imaging modality that can potentially offer a solution for the diagnosis of COVID-19 cases. In this work, we examined eleven deep convolutional neural network architectures for the task of classifying chest X-ray images as belonging to healthy individuals, individuals with COVID-19 or individuals with viral pneumonia. All the examined networks are established architectures that have been proven to be efficient in image classification tasks, and we evaluated three different adjustments to modify the architectures for the task at hand by expanding them with additional layers. The proposed approaches were evaluated for all the examined architectures on a dataset with real chest X-ray images, reaching the highest classification accuracy of 98.04% and the highest F1-score of 98.22% for the best-performing setting.

## 1. Introduction

The 2019 novel corona virus (COVID-19) pandemic that was first reported in Wuhan, China, in December 2019, has become a public health issue around the world [1]. The infection that caused the COVID-19 pandemic was called a Severe Acute Respiratory Syndrome, also known as SARS-CoV-2 [2]. As of the second quarter of 2021, the COVID-19 pandemic keeps on affecting the well-being and health of the general public. A critical step in the battle against COVID-19 is a reliable and effective detection method for diagnosing infected patients, with the end-goal of prompt treatment and care. Corona viruses are a huge group of viruses that cause illness. Examples are Middle East Respiratory Syndrome (MERS-CoV), Severe Acute Respiratory Syndrome (SARS-CoV) and COVID-19. COVID-19’s earliest symptoms include, fever, cough, fatigue or myalgia [3,4,5].

The main screening technique used for identifying COVID-19 cases is reverse transcription polymerase chain reaction testing (RT-PCR) [6,7], which can recognise SARS-CoV-2 RNA from respiratory samples gathered through various means, e.g., nasopharyngeal or oropharyngeal swabs. Initial findings have stated that RT-PCR testing shows relatively poor sensitivity [8]. Further findings showed that RT-PCR testing is highly specific and the probability of false positives is low. However, the amount of virus in a swab varies among patients, so at the initial test, it can provide a true negative result that turns out to be a false negative at a later stage, which is dangerous [9,10]. A screening method that can additionally be used for COVID-19 detection is radiography assessment, where chest radiography imaging such as computed tomography (CT) or chest X-ray (CXR) is conducted and analysed by radiologists to search for visual markers related to SARS-CoV-2 viral infection. Early investigations showed that patients present abnormalities in chest radiography images that pointed out features of those with COVID-19 [11,12], with some recommending that radiography assessment could be utilised as a primary tool for diagnosing COVID-19 [13]. However, the American College of Radiology (ACR) [14] and the World Health Organisation (WHO) [15] are sceptical and urge caution in using chest X-rays or chest CT scans as a primary diagnostic tool for COVID-19, with the WHO suggesting the use of chest imaging if RT-PCR is not available at all or in a timely manner.

Studies have shown that patients with COVID-19 exhibit some characteristics on their chest X-rays: It primarily affects the peripheral and lower areas of the lungs and presents nodular shadowing, ground glass opacity and accumulations of fluid and tissue in pulmonary alveoli, which is also called consolidation [16,17]. An important observation made during a study of COVID-19-related imaging diagnosis is that the initial symptoms are not visible at all or are slightly visible on chest X-rays within the first three days from symptom onset, but they are very obvious after 10 to 12 days [18]. A common complication of influenza-like illnesses is viral pneumonia, which has also been shown to be a complication of COVID-19 [19]. Medical imaging, specifically chest computed tomography (CT) and chest X-ray, is frequently utilised as an integrated assessment in the detection and management of pneumonia. However, given that viral pneumonia can be a complication of various illnesses, it is important to be able to assess whether a specific case is related to COVID-19.

The required medical and clinical resources for COVID-19 diagnosis at a global scale are a major challenge. Several countries are unable to carry out large numbers of COVID-19 tests [20], because of limited diagnosis tools. There is a need to identify a quick and reliable tool that can detect COVID-19 effectively with minimal effort. Numerous attempts have been conducted to devise an appropriate and quick approach to recognise infected patients at an early stage. After taking chest CT scans of 21 patients with COVID-19 in China, Guan et al. [21] found that CT scan analysis showed reciprocal pulmonary parenchymal abnormalities and pneumonic consolidation, as well as a fringe lung distribution. Thus, the analysis effectively extracted the main features of the virus.

All things considered, radiography assessment is quicker and has more prominent accessibility than RT-PCR testing, given the availability of chest radiology imaging systems in the healthcare sector. In addition, the turnaround time for X-ray examination is approximately 5.08 h [22]. Chest X-ray imaging is frequently used as a standard testing technique for respiratory complaints [23] and is easily accessible, making it a suitable COVID-19 detection method. Nevertheless, considering that COVID-19 symptoms are not visible in X-rays during the first days of the infection [18], chest X-ray imaging cannot fully replace RT-PCR, but can play an important role in patient screening to indicate potential COVID-19 cases, especially when RT-PCR is not easily accessible. However, probably the greatest bottleneck confronted is the requirement of expert radiologists to decipher the radiography images since the visual pointers can be unobtrusive. Consequently, computer diagnostic frameworks that can help radiologists quickly and precisely decipher radiography images to recognise COVID-19 cases are of critical importance for an accessible-to-all protect against the virus.

The critical need to develop solutions to help curb the challenges in the effort against COVID-19, motivated by the availability of CT and chest X-ray images of COVID-19 cases, led this study to carry out experiments on deep convolutional neural network (CNN) architectures that can effectively detect COVID-19 with the highest accuracy. To this end, we opted to select eleven well-established CNN architectures that have been shown to be efficient in various image classification tasks and conducted a comparative study to examine their performance on the task of classifying chest X-ray images as belonging to healthy individuals, individuals with COVID-19,or individuals with non-COVID-19-related viral pneumonia. Three different versions of the examined CNN architectures were evaluated: (1) a baseline version where the pretrained CNN models were used as is, changing only the classifier in their output to suit the task at hand; (2) a modified version with two additional fully connected layers between the convolutional base and the classifier and dropout layers before each fully connected layer; and (3) a modified version with two larger fully connected layers between the convolutional base and the classifier and batch normalisation and leaky ReLU layers before each fully connected layer. All the examined approaches were trained and evaluated on a dataset with real chest X-ray images using a stratified five-fold cross-validation procedure, while the best-performing model was also evaluated on a completely unseen dataset. Supervised classification experiments demonstrated the efficiency of the proposed approach, reaching the highest classification accuracy of 98.04% and the highest F1-score of 98.22% for the best-performing setting.

The novelty of this work can be summarised as follows: (a) We provide a comparative study of the performance of multiple well-established CNN architectures that have been proven to work well on generic image classification tasks and examine them on the task of classifying chest X-ray images as belonging to healthy individuals, individuals with COVID-19 or individuals with non-COVID-19-related viral pneumonia. (b) We propose two different adjustments of these architectures and examine how classification performance is affected on the examined task. (c) We examine the performance of the models when using weights pretrained on ImageNet without any additional training and when end-to-end training is applied. (d) We show that although the pretrained CNN models have been proven to be very efficient on generic image classification tasks (e.g., ImageNet), performance suffers when fine-tuned for chest X-ray image classification, but minor extensions of the architectures, such as the ones proposed in this work, allow these CNN architectures to perform exceptionally well on the task, while also exploiting the available pretrained weights, thus reducing the amount of X-ray images needed for training the models. (e) Finally, similar available works in the literature commonly evaluate the performance of the proposed models by dividing their dataset into a training set and a test set. However, due to the limited availability of COVID-19-related images, such datasets are typically created by combining multiple datasets, which can lead to the trained neural networks learning features that are specific to the dataset better than the ones that are specific to the disease, thus leading to overfitting and reduced generalisation ability [24]. To ensure that the models proposed in this work do not suffer from this issue, in addition to our test set, we evaluated the performance of our models on a completely independent dataset, without any additional training or fine-tuning.

The rest of this paper is organised into five sections. Section 2 provides a brief literature review on the use of deep CNN architectures for medical image classification. Section 3 describes the proposed methodology, while the experimental results are presented and discussed in Section 4. Finally, conclusions are drawn in Section 5.

## 2. Related Work

The use of deep learning has proven to be very effective and reliable in revealing features, which are not evident, in images. Deep learning is currently widely used in the medical field for image classification and the detection of human diseases through computer-aided diagnosis [25,26,27,28]. Convolutional neural networks (CNNs) have demonstrated beneficial learning and useful feature extraction capabilities, and thus have been embraced by many researchers [29]. The use of pretrained networks on labelled medical images to train CNNs on disease classification has been proven to result in high performance, suggesting that in some cases, CNNs have achieved a level that is equivalent to or even better than certified human radiologists [30,31,32]. CNNs have been applied to recognise pulmonary nodules or masses from CT images [33], on chest X-ray images for the diagnosis of pneumonia [34], for cystoscopic image recognition extraction [35], for automated detection of polyps during colonoscopy [36], etc.

Transfer learning has also proven to be very efficient in deep learning applications, making it possible to use pretrained networks from different applications in order to save time and power in training a model to achieve high performance. This concept was utilised by Vikash et al. [37] in pneumonia detection utilising preprepared models trained on the ImageNet [38] dataset. Xianghong et al. [39] modified the VGG16 model and used it for identification of lung regions and classification of various kinds of pneumonia. Ronneburger et al. [40] implemented a CNN with a small set of images, but applying a data augmentation technique to attain a better result. They applied the U-net to a cell segmentation task on two datasets, namely “PhC-U373” [41] and “DIC-HeLa” [42], and achieved an average IOU score of 92% and 77.5%, respectively. Ho et al. [43] reported an accurate identification of 14 thoracic diseases using feature extraction techniques and the pretrained DenseNet-121 [44] model. Lakhani et al. [31] also carried out an experiment on pulmonary TB detection using GoogLeNet [45] and AlexNet [29] by applying image augmentation techniques and attained an area under the curve (AUC) accuracy of 99%.

Wang et al. [46] carried out an experiment using chest X-ray data, labelled with eight diseases, and trained a deep CNN model by utilising weight parameters from VGGNet-16 [47], AlexNet [29], ResNet-50 [48] and GoogLeNet [45]. ResNet-50 showed better results than other models in the classification of seven diseases except for one, for which AlexNet performed better. Some of the AUC scores were as follows: “Cardiomegaly” (81.41%), “Pneumothorax” (78.91%), “Effusion” (73.62%), “Nodule” (71.64%), “Atelectasis” (70.69%).

Wang et al. [49] utilised deep learning methods on CT images to detect COVID-19 with a sensitivity, specificity and accuracy of 87%, 83% and 89.5%, respectively. Narin et al. [50] carried out an experiment on chest X-ray images, using Inception-ResNetV2 [51], InceptionV3 [52] and ResNet-50 [48] for the classification of COVID-19 and normal images. The ResNet50 model achieved the best classification accuracy of 98%, while for InceptionV3 97% and Inception-ResNetV2 87%. Wang et al. [53] presented a deep learning CNN architecture, called COVID-Net, for COVID-19 detection from chest X-rays, achieving a 92.4% accuracy.

Chowdhury et al. [54] carried out two COVID-19-related experiments with a chest X-ray dataset. The first utilised two classes, normal and COVID-19, while the second utilised three classes, namely COVID-19, viral pneumonia and normal. They experimented using transfer learning with and without image augmentation and tested and validated their approach using eight pretrained networks. Classification accuracy reached 99.41% for the two-class problem without image augmentation and 99.70% with image augmentation, while for the three-class problem, classification accuracy reached 97.74% without image augmentation and 97.94% with image augmentation.

The use of chest X-rays for COVID-19 detection has been the focus of multiple other recent studies. Shibly et al. [55] proposed the use of VGG16 [47] and the Faster R-CNN framework to detect COVID-19 from chest X-rays, achieving an accuracy of 97.36%, a sensitivity of 97.65% and a precision of 99.28%. Jain et al. [56] used the ResNet101 model, achieving a 98.93% accuracy, 98.93% sensitivity, 98.66% specificity, 96.39% precision and 98.15% F1-score. Nishio et al. [57] proposed a chest X-ray-based computer-aided diagnosis (CADx) system for classification into COVID-19 pneumonia, non-COVID-19 pneumonia and normal. They experimented using the VGG16 [47], MobileNet [58], DenseNet-121 [44], and EfficientNet [59] CNN models and reported that VGG16 performed best with an accuracy of 83.6%. Similarly, Apostolopoulos et al. [60] experimented with the VGG19 [47], MobileNetv2 [58], Inception [52], Xception [61] and InceptionResNetv2 [51] CNNs, reporting that MobileNetv2 performed best with an accuracy of 96.78%, a 98.66% sensitivity, and a 96.46% specificity. Sahlol et al. [62] attempted to reduce the computational complexity of CNN-based approaches by combining CNN-based features with the marine predators algorithm for swarm-based feature selection. Experiments on two different chest X-ray datasets demonstrated a maximum accuracy of 98.7%.

Apart from X-rays, other approaches have also been employed for COVID-19 detection. For example, considering that a cough is a vital symptom of COVID-19, Chuma et al. [63] carried out an experiment on a cough classification task using a K-band continuous-wave Doppler radar sensor and the AlexNet [29], VGG-19 [47] and GoogLeNet [45] CNN architectures, reporting that AlexNet performed best, with an accuracy of 88% when people were 1 m away from the sensor, 80% for 3 m and 86.5% for mixed 1 m and 3 m data.

## 3. Methodology

In this work, we examined various deep neural network architectures for the task of classifying chest X-ray images as belonging to healthy individuals, individuals with COVID-19 or individuals with viral pneumonia (non-COVID-19-related) and proposed three different adjustments to the architectures that led to increased performance for the task at hand. We opted to include viral pneumonia cases that were not related to COVID-19 as the third class in our experiments, since viral pneumonia is a complication of various diseases, but has been shown to also be a complication of COVID-19 [19]. The performance of the proposed approaches was evaluated on a publicly available chest X-ray image dataset [54], demonstrating their efficiency in improving COVID-19 detection regardless of the base network architecture used.

### 3.1. Dataset

**Figure 1:**
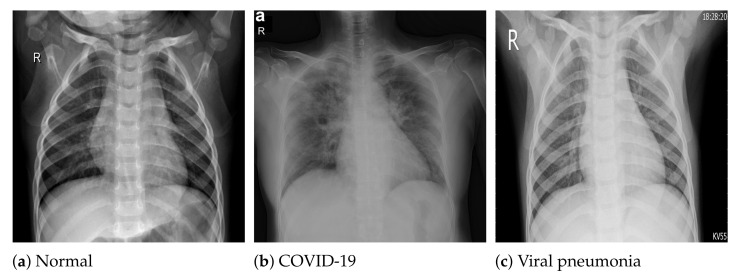
Sample X-ray images from the used dataset.

The COVID-19 radiography database [54] was selected for this work. This database was compiled by a team of researchers from the University of Doha in Qatar and the University of Dhaka in Bangladesh, who collaborated with medical doctors from Pakistan and Malaysia to create a database of chest X-ray images for COVID-19-positive cases along with normal and viral pneumonia images. The database consists of 2905 chest X-ray images, including 219 COVID-19-positive images, 1341 normal images and 1345 viral pneumonia images. The images in the COVID-19 radiography database were collected from various sources, including the Italian Society of Medical and Interventional Radiology (SIRM) COVID-19 database [64], Cohen et al.’s COVID-19 image data collection [65], the ChestX-ray8 database [46], the Kermany et al. [66] pneumonia chest X-ray images dataset, as well as some online repositories [54], where physicians and researchers have uploaded COVID-19-related chest X-ray images. All the images are stored in Portable Network Graphics (PNG) file format (24 bit RGB), with a resolution of 1024×1024 pixels. Figure 1 depicts sample images from the database for COVID-19, normal and viral pneumonia chest X-ray images.

### 3.2. Data Augmentation

One of the obstacles when attempting to apply deep learning techniques to solve a problem is the lack of sufficiently large amounts of data for training the deep learning models. Depending on the application and field, acquiring more data can be very arduous and costly, both in terms of time and resources. Data augmentation, i.e., increasing the amount of available data without gathering new data by applying various operations on the available data, has proven to be effective in image classification [67]. The technique has been used in the ImageNet classifier challenge by those that won the competition [29,48], and it is widely used by researchers to increase the training data, thereby avoiding overfitting [68].

In this work, we opted to use data augmentation techniques because of the limited number of images in the COVID-19 radiography database, especially for the COVID-19 class, which contained only 219 samples. To achieve this, the images in the training set at each training fold of the cross-validation procedure were used to create additional images by: randomly flipping them horizontally, randomly flipping them vertically, rotating at a random angle between −90 and 90 degrees, randomly shifting across the width by 10% of the total width, randomly shifting across the height by 10% of the total height, randomly zooming within a range of 0.9 to 1.1, randomly shearing in the counterclockwise direction by an angle of 0 to 0.1 rad, randomly shifting the brightness between 0.5 and 1.5, and finally, rescaling by a factor of 1/255. All pixels outside the boundaries of the input were filled using the nearest neighbour approach.

It must also be noted that all random values for the data augmentation operations were generated using a uniform probability distribution and that the Keras ImageDataGenerator class was used for the real-time creation of batches of augmented images during each training procedure. By using this data augmentation technique, the number of images in the training set was significantly increased, allowing the efficient use of deep learning techniques by training the machine learning models using a much larger amount of training images. Furthermore, it must be noted that the augmented images were only used for training the models and not for testing; thus, only original images from the dataset were used for testing the trained models.

### 3.3. Classification Using Deep Neural Networks

Considering the limited amount of available images for the task at hand, we opted to base our approach on established architectures that have been proven to be efficient feature extractors for object detection applications and have been trained using sufficiently large image datasets. To this end, we selected eleven well-established CNN architectures that have achieved state-of-the-art results and were pretrained on the ImageNet [38] dataset, which consists of 1.4 million labelled images with 1000 classes. The selected deep learning models are very popular and widely used in computer vision tasks and have proven to excel in image classification problems. The following deep convolutional neural networks were examined for the task of classifying COVID-19, normal and viral pneumonia X-ray images (3-class problem): EfficientNetB4 [59], EfficientNetB7 [59], VGG16 [47], Xception [61], InceptionResNetV2 [51], InceptionV3 [52], MobileNetV2 [58], ResNet50V2 [48], DenseNet121, DenseNet169 and DenseNet201 [44]. The following three different approaches were used to adjust these architectures to the examined task, which were all trained using the Adam optimiser, a batch size of 16 and a learning rate of 0.0001. Furthermore, cross-entropy was used as the loss function, computed as $L_{CE}=-\sum_{c=1}^{M}y_{o,c}\log\left(p_{o,c}\right)$, with M=3 the number of classes, $y_{o,c}$ a binary indicator (0 or 1) if observation *o* belongs to class *c* and $p_{o,c}$ the predicted probability that observation *o* is of class *c*.

**Figure 2:**
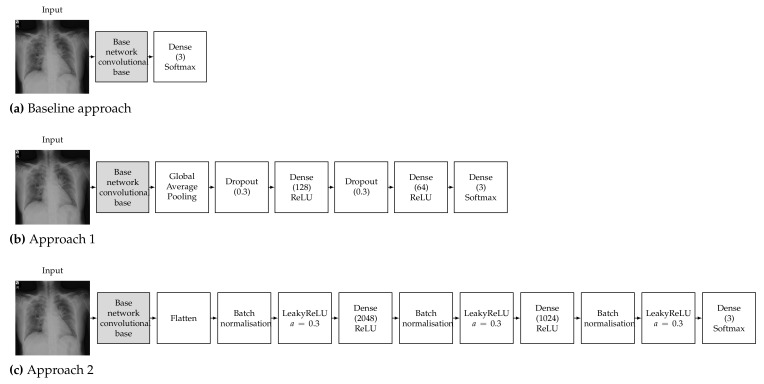
Proposed neural network architecture for the (**a**) baseline approach, (**b**) Approach 1 and (**c**) Approach 2.

To train a robust CNN that will accurately classify images, hyperparameters must be tuned according to the examined problem. Our choice of hyperparameters was made after some preliminary experimentation and by following the conclusions of the study of Kandel et al. [69] on the effect of the batch size and learning rate when the Adam optimiser is used to train CNNs for medical image classification, which recommended that decreasing the batch size and lowering the learning rate will allow the network to learn better and generalise more accurately. It must also be noted that Keras and TensorFlow 2 were used for all the experiments; thus, all pretrained networks used in this work refer to the respective Keras implementations. Detailed diagrams of the proposed neural network architectures are depicted in Figure 2.

#### 3.3.1. Baseline Approach

The pretrained models were used as a feature extractor. CNNs are made up of two parts, which are the convolutional base and the classifier. The convolutional base contains the convolutional and pooling layers, which extract features from images. The classifier part is composed of a fully connected layer (softmax) with the goal of classifying images based on detected features. The concept of the baseline approach is to make use of only the convolutional base (feature extractor) without the classifier and feed its output directly into a softmax-activated layer [70] with 3 neurons (Figure 2a), corresponding to the number of targeted classes (COVID-19, normal, viral pneumonia). The convolutional base layer was set to freeze, in order to take advantage of the features learned by the models trained on the ImageNet dataset; therefore, the weights of the pretrained network were not updated during training. Then, the new classifier was trained to determine one of the three available classes given the set of extracted features [71]. It must be noted that contrary to the other examined architectures, VGG16 contains two fully connected layers before the final classifier. We opted to keep these layers in the VGG16 baseline model in order to be consistent with changing only the final softmax-activated layer for all the examined architectures.

#### 3.3.2. Approach 1

The deep learning classification approach used is called round-off fine-tuning of the entire model. As shown in Figure 2b, for Approach 1, we added a new classifier with a new mini network of two small fully connected layers that fit our purpose. The first fully connected layer had 128 neurons, while the second had 64 neurons, followed by a softmax classifier with 3 neurons corresponding to our 3 output classes. We also added a global average pooling layer after the last convolutional block of the base network and a dropout layer with a rate of 0.3 before each of the two intermediate dense layers, which has been proven to help reduce the risk of overfitting [72]. Then, the pretrained weights on ImageNet were used as the initialisation of the base network in order to adapt the pretrained features to the new data.

#### 3.3.3. Approach 2

The second approach is one of the most commonly used fine-tuning methods for image classification. We added a new classifier with two fully connected layers. The first fully connected layer had 2048 neurons, while the second had 1024 neurons, followed by a softmax classifier with 3 neurons corresponding to our 3 output classes. As shown in Figure 2c, we also added a flatten layer after the output of the convolutional base of the base network and a batch normalisation layer before each of the three dense layers, which has also been proven to help reduce the risk of overfitting and accelerate the learning process [73]. We also opted to add a leaky ReLU layer after each batch normalisation layer, as this has be proven to improve the performance of a network [74]. Then, similar to Approach 1, the pretrained weights on ImageNet were used as the initialisation of the base network in order to adapt the pretrained features to the new data.

### 3.4. Hyperparameter Settings and Added Layers

Given the countless choices in the numbers, types and parameters of layers, we opted to examine the performance of architectures that were as simple as possible, adding only 3 dense layers, and compared them with a simpler approach (Approach 1) and a more complex one (Approach 2). The hyperparameters and added layers of the proposed approaches were selected by conducting some preliminary experiments on a smaller dataset, as follows: For all approaches, we opted to use the Adam optimiser, a batch size of 16 and a learning rate of 0.0001, as these settings performed best in a study carried out by Kandel et al. [69] on the effect of batch size and the impact of learning rates on the performance of CNNs for image classification of medical images. In addition, three fully connected (dense) layers were used after the convolutional layers of the base network for both Approaches 1 and 2. In both cases, the second dense layer had half the neurons of the first one, while the third dense layer (output layer) had three neurons corresponding to the three output classes.

For the dropout layers in Approach 1, a dropout rate of 0.3 was used. Dropout was proposed by Hinton et al. [72] as a regulariser that randomly sets a portion of the activations to the fully connected layers to zero during training, leading to improved generalisation ability and largely preventing overfitting [75]. Apart from the dropout layers, global average pooling was used in Approach 1 to generate a feature map from the output of the convolutional layers of the base network. It is a structural regulariser that helps to avoid overfitting in this layer, first proposed by Lin et al. [70].

Batch normalisation was used in Approach 2 to normalise activations in intermediate layers of the architecture, as it has been shown to improve accuracy, reduce the risk of overfitting and speed up the training process of deep neural networks [76]. Furthermore, a flatten layer was used in order to convert the multidimensional output of the convolutional layers of the base network into a one-dimensional vector, which can be fed into the fully connected layer [77]. Finally, Approach 2 used leaky ReLU activation layers (α=0.3), as they have been shown to improve the performance of a network by Wang et al. [74], who compared the performance of three different activation functions.

### 3.5. Label Smoothing

Label smoothing is a regularisation technique that addresses both overfitting and overconfidence problems. It is a simple method that makes a model more robust and enables it to generalise well. When cross-entropy is used as a loss function, the training process aims to minimise $L_{CE}=-\sum_{c=1}^{M}y_{o,c}\log\left(p_{o,c}\right)$, where $y_{o,c}$ is a binary indicator (0 or 1) showing whether observation *o* belongs to class *c*. In this case, $y_{o,c}$ is considered a hard target as it is either 0 or 1. When label smoothing is used, the targets $y_{o,c}$ are modified as $y_{o,c}^{LS}=y_{o,c}\left(1-\alpha \right)+\frac{\alpha }{M}$, with *M* being the number of classes and α the label smoothing parameter. Szegedy et al. [52] proposed the label smoothing technique, which improved the performance of the Inception architecture on the ImageNet dataset, and several other state-of-the-art deep learning classification models have adopted this method since [78,79]. In this work, we adopted label smoothing to improve the performance of our models by minimising cross-entropy using soft targets instead of hard targets, with a smoothing parameter α=0.1, thus encouraging the model to be less confident and leading to better generalisation.

## 4. Results and Discussion

The performance of the eleven examined deep convolutional neural network models for the there-class problem (normal, COVID-19, viral pneumonia) using the baseline and the other two proposed approaches was evaluated by conducting supervised classification experiments. Approach 1 and Approach 2 were evaluated twice, once keeping the pretrained weights of the base networks frozen and only training the additional layers and once using the pretrained weights as the initialisation and training the networks end-to-end. A stratified five-fold cross-validation procedure was followed in order to provide a fair estimate of the classification performance and avoid overfitting. To this end, the available images in the COVID-19 radiography database were divided into five groups, respecting the class distribution, and at each fold of the cross-validation procedure, one group was used for testing and the rest for training the examined models. This process was repeated until all groups had been used for testing, and the overall classification performance was computed by averaging the performance across the five folds.

The computed performance metrics were the accuracy, F1-score, precision and recall. Furthermore, since the F1-score, precision and recall depend on which class is considered as positive, their reported scores in this work are the average scores among the three examined classes. In addition to these four metrics, the Jaccard index and the Dice coefficient were also computed from the aggregated test groups across the five folds of the five-fold cross-validation procedure. All experiments were conducted by employing the TensorFlow library and the Keras API, using the Python programming language on the Google Colab Pro platform (Nvidia Tesla T4 and P100 GPU, 24 GB RAM). It must also be noted that the chest X-ray images were resized to 300×300 pixels before being fed as input to the examined network models, since this size was close to the input size for which they were originally designed. Given that the examined networks expected different input sizes and to achieve a fair comparison, we opted to resize the images to a common size that was close to the one expected by the networks, which varied from 224×224 to 456×456 pixels.

### 4.1. Results for the Baseline Approach

The classification performance achieved using the baseline approach and the two other proposed approaches is reported in Table 1, Table 2 and Table 3, respectively, in terms of the classification accuracy, F1-score, precision, recall, Jaccard index and Dice coefficient metrics. Table 1 contains the results for the baseline approach, which performed the worst among the examined approaches. The results were quite stable across all the proposed models, with an average F1-score within the range of 76.39–91.94%. VGG16 performed the best across all metrics, achieving the highest average F1-score of 91.94%, while EfficientNetB7 achieved the lowest average F1-score of 76.39%. The slightly higher performance of the VGG16 architecture compared to the others can be attributed to the additional two fully connected layers before the final softmax-activated layer, as explained in Section 3.3.1. Indeed, when performing the same experiment for VGG16 without the two fully connected layers, the F1-score dropped to 87.13%.

### 4.2. Results for Approach 1

Results for Approach 1 are reported in Table 2. From Table 1, Table 2 and Table 3, it is evident that Approach 1 with end-to-end training provided the best performance among the examined approaches, achieving considerably high metric values for all the examined models. In the case of end-to-end training, the EfficientNetB4- and Xception-based models achieved the highest average F1-scores of 98.22% and 98.20%, respectively, while the VGG16 and InceptionV3-based models achieved the lowest average F1-scores of 95.39% and 95.76%, respectively. On the other hand, the rest of the models, namely EfficientNetB7, ResNet50V2, InceptionResNetV2, MobileNetV2, DenseNet201, DenseNet169 and DenseNet121 models, achieved an average F1-score within the range of 96.66–97.92%. In the case of using the pretrained weights for the base network, performance suffered considerably compared to end-to-end training, with ResNet50V2 achieving the best performance across all metrics, with an F1-score of 90.81%. Comparing the results from Table 1 and Table 2, it is evident that the use of Approach 1 led to significant improvement in classification performance.

### 4.3. Results for Approach 2

Table 3 contains the classification results for Approach 2, which consistently performed slightly worse than Approach 1, as can be seen from Table 2 and Table 3. In the case of end-to-end training, similar to Approach 1, the performance for Approach 2 was quite stable across the examined models, with an average F1-score within the range of 94.83–97.27%. The InceptionV3 and Xception models achieved the highest average F1-scores of 97.27% and 97.26%, respectively, while VGG16 achieved the lowest F1-score of 94.83%. In the case of using the pretrained weights for the base network, the performance decreased marginally compared to end-to-end training, with DenseNet169 and DenseNet121 achieving the highest F1-scores of 96.12% and 96.06%, respectively, with VGG16 achieving the lowest F1-score of 89.22%.

### 4.4. Validation on an Unseen Dataset

To further evaluate the generalisation ability of the proposed approach, we examined the classification performance of our best-performing model, i.e., the EfficientNetB4-based Approach 1, on an unseen dataset without any additional training or fine-tuning. To this end, we used the COVID-19 Image Repository (Version 2.0) [80], which contains 243 chest X-ray images of COVID-19 cases from the Institute for Diagnostic and Interventional Radiology, Hannover Medical School, Hannover, Germany. Considering that the available COVID-19 X-ray datasets are commonly collections of images from various sources, we selected this dataset in order to ensure that no overlap existed between its images and the images used for training our models (Section 3.1). As shown in Table 4, the EfficientNetB4-based Approach 1 model was able to correctly classify 234 out of the 243 (96.30%) COVID-19-related images, misclassifying 7 images as normal (2.88%) and 2 images as viral pneumonia (0.82%). These results on the unseen dataset that was not used for training or fine-tuning our model further demonstrated its efficiency and generalisation ability.

### 4.5. Execution Time

Information regarding the size in terms of trainable parameters and the time and number of epochs taken to train the best-performing models for the baseline approach, Approaches 1 and 2 with end-to-end training and Approaches 1 and 2 using the frozen pretrained weights for the base network is provided in Table 5. The average execution times were measured using TensorFlow and the Keras API on the Google Colab Pro platform (Nvidia Tesla T4 and P100 GPU, 24 GB RAM), as well as the training parameters described in Section 3.3. From Table 5, it is evident that the fastest model to train per epoch was VGG16 for the baseline approach (108 s/epoch), followed by the EfficientNetB4 and Xception for Approach 1 with end-to-end training (112 and 111 s/epoch, respectively), which also achieved the best overall classification performance. DenseNet169 for Approach 2 using the pretrained weights for the base network required the most time to train at 166 s/epoch. However, it must be noted that for the sake of consistency and fairness, the training parameters were the same for all the models tested. Consequently, fine-tuning the parameters for each specific model could potentially lead to better overall training times for some of the models, and as a result, the execution times and number of epochs required for training that are reported in Table 5 must be taken into consideration only under the specific configuration and hardware.

### 4.6. Discussion

**Figure 3:**
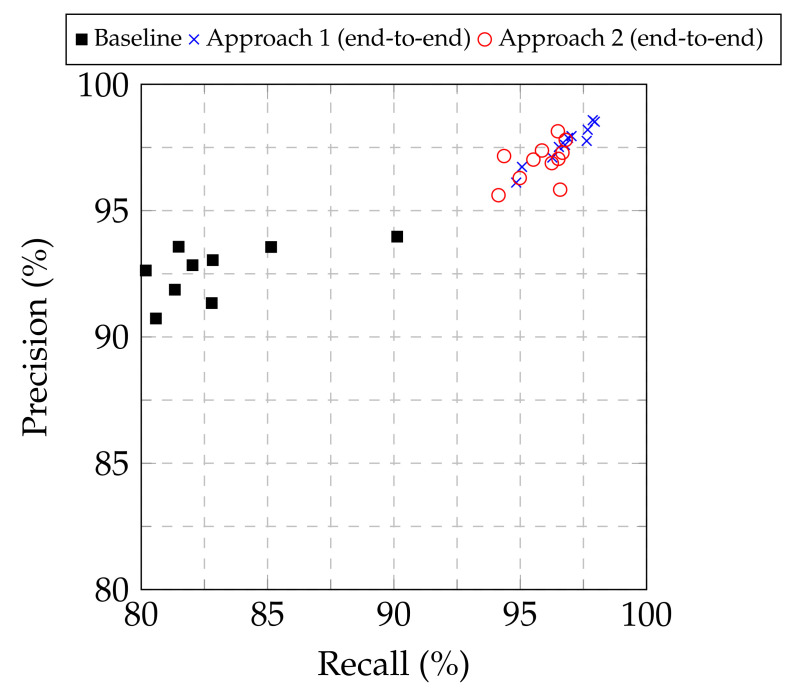
Precision vs. recall for the baseline approach and Approaches 1 and 2 for end-to-end training.

From Table 1, Table 2 and Table 3, as well as from the the precision–recall plot in Figure 3, it is evident that both Approach 1 and Approach 2 led to improved performance compared to the baseline approach for all the examined base models. Furthermore, Approach 1 consistently provided higher classification performance, in terms of the F1-score, among all the approaches examined, regardless of the base model used. Consequently, Approach 1 demonstrated its superiority to modify pretrained image classification deep CNN models to classify chest X-ray images into normal, COVID-19 and viral pneumonia. Regarding the optimal base CNN model, the results were not conclusive for selecting a single model, but showed two out of the eleven examined candidates as suitable. The EfficientNetB4- and Xception-based models provided similar results for Approach 1 in terms of accuracy (98.04% vs. 98.00% respectively), F1-score (98.22% vs. 98.20%), precision (98.52% vs. 98.58%) and recall (97.95% vs. 97.87%), with the EfficientNetB4-based model being marginally better in most cases, while also achieved a marginally higher Jaccard index (96.52% vs. 95.98%) and Dice coefficient (98.23% vs. 97.95%). As a result, both models can be considered as suitable for the examined task.

**Figure 4:**
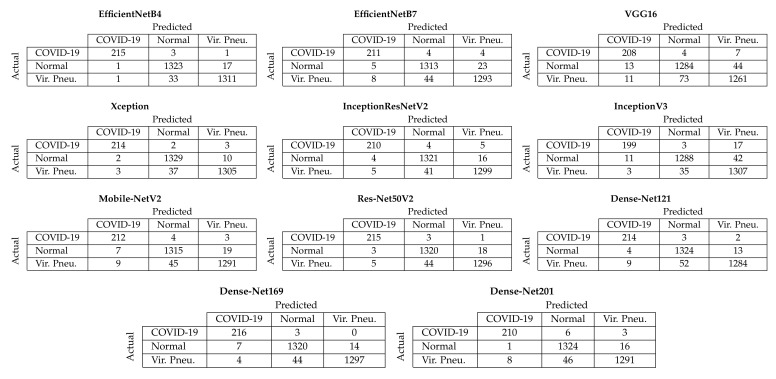
Aggregated confusion matrices for Approach 1 (end-to-end training).

Figure 4 depicts the aggregated confusion matrices, i.e., the sum of the confusion matrices from each fold of the five-fold cross-validation procedure, for Approach 1 (end-to-end training). It must be noted that since the additional images created through data augmentation (Section 3.2) were only used for training, the testing sets contained only the original images; thus, the number of samples for each class in the confusion matrices is equal to the number of samples per class in the dataset (Section 3.1). From these confusion matrices, it is evident that the two best-performing models achieved almost similar performance in correctly classifying COVID-19 samples, with the EfficientNetB4-based model correctly classifying 215/219 COVID-19 samples and the Xception-based model 214/219. Despite other models also achieving similar performance for the COVID-19 samples, the EfficientNetB4- and Xception-based models for Approach 1 achieved the best balance across all three available classes.

The EfficientNetB4 model belongs to the EfficientNet family, which consists of eight models, ranging from B0 to B7, and has been shown to achieve both higher accuracy and better efficiency than previous ConvNets, with a reduced parameter size. This group of models was developed by Google AI researchers and was scaled down by balancing the depth, width and resolution, which has led to effective results, and it is also smaller and faster than existing deep learning models [59]. More specifically, EfficientNetB4 achieved state-of-the-art 83.0% top-1/96.3% top-5 accuracy on ImageNet and has a size of 75 MB with over 19 million parameters [59]. The Xception model has previously achieved 79.0% top-1/94.5% top-5 accuracy on ImageNet and has a size of 88 MB with over 22 million parameters [61].

Interestingly, when using the pretrained weights for the base networks, Approach 2 outperformed Approach 1 (maximum F1-score of 96.12% for DenseNet169 vs. 90.81% for ResNet50V2, respectively). Despite performing worse than Approach 1 with end-to-end training, it seems that the more complex architecture of Approach 2 led to a better use of the pretrained features compared to the simpler architecture of Approach 1, when no end-to-end training was applied.

To further demonstrate the performance of the proposed approach, we compared the results of the best model for each approach (EfficientNetB4-based Approach 1 and InceptionV3-based Approach 2, both with end-to-end training) to other works in the literature that used the same dataset for the same classification task (COVID-19 vs. normal vs. viral pneumonia), as shown in Table 6. Given the large number of chest X-ray datasets in the literature and the fact that many works combine multiple datasets to increase the number of available samples, we opted to include in our comparison only works that used the exact same dataset as this work, in order to provide a fair comparison. From Table 6, it is evident that the proposed approach achieved a higher F1-score (98.22%) compared to the other methods, except for the CNN+BiLSTM approach of Aslan et al. [81], which achieved a marginally higher F1-score (98.76%) by utilising the more computationally complex BiLSTM layers on top of the CNN layers.

**Figure 5:**
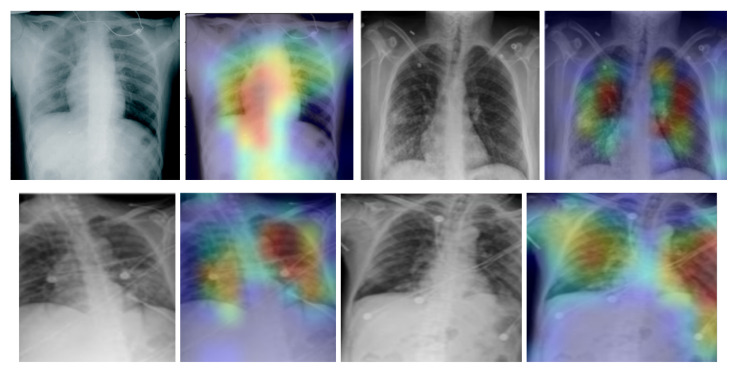
Grad-CAM visualisation for the COVID-19 class using the EfficientNetB4-based Approach 1 model for four chest X-ray images.

In addition, we used the gradient-weighted class activation mapping (Grad-CAM) method to visualise class activation heat maps for the best-performing model (EfficientNetB4-based Approach 1), as shown in Figure 5 for four COVID-19-positive images. The Grad-CAM method uses the gradients of any target class in a classification network flowing into the final convolutional layer to produce a coarse localisation map that highlights the most important image regions for predicting the specific class. It is based on the CAM method, which finds the discriminative regions for a CNN prediction through the computation of class activation maps, which assign importance to every position (i,j) in the last convolutional layer by computing the linear combination of the activations, weighted by the corresponding output weights for the observed class. Grad-CAM extends the CAM method by incorporating gradient information in the computation of the class activation maps (heat maps). By using the heat maps from the Grad-CAM method, we can examine the regions within the input image on which the CNN model focuses to make the decision for each class. By examining the examples in Figure 5 for our best-performing model, it is evident that the trained CNN model focuses on the areas of the lungs, as expected, and thus, we can be confident that the model relies on features extracted from the image regions that contain the information regarding COVID-19, viral pneumonia or healthy lungs, and not on information related to the images, to artefacts in the images or to the source of the images.

Regarding the applicability of our work in clinical practice, research has shown that the severity of COVID-19-related findings on chest X-rays peaks after 10–12 d from the initial onset of symptoms, whereas they are not visible or are slightly visible during the first 3 d [18]. Consequently, the proposed models could assist with COVID-19 diagnosis after symptom onset. However, it must be noted that the American College of Radiology (ACR) [14] and the WHO [15] urge caution in using chest radiography as a primary diagnostic tool for COVID-19, with the WHO suggesting its use in cases in which RT-PCR is not available at all or in a timely manner. In addition, despite the high classification performance of the proposed models on images from various sources, a larger study that would include numerous COVID-19-positive chest X-rays, acquired from multiple radiography devices at multiple stages of the disease, would be required to evaluate their suitability for real-world clinical practice. Nevertheless, the acquired results are very promising, demonstrating how deep learning image classification models could potentially provide crucial help on the diagnosis of COVID-19.

## 5. Conclusions

The global COVID-19 pandemic that started in 2019 has demonstrated the need for quick, inexpensive, accessible and reliable diagnostic methods for detecting infected individuals. In this work, we evaluated the performance of eleven deep convolutional neural network architectures for the task of classifying chest X-ray images as belonging to healthy individuals, individuals with COVID-19 or individuals with viral pneumonia. The eleven examined CNN models were selected due to their proven efficiency in image classification tasks and were modified in order to be adjusted for the task at hand. Supervised classification experiments using a five-fold cross-validation procedure were performed in order to evaluate the performance of three different modifications of the examined base CNN models on a dataset with real chest X-ray images that contained normal images, COVID-19-positive images and viral pneumonia images. The EfficientNetB4- and the Xception-based models, using Approach 1 and end-to-end training, provided the best classification performance, reaching an accuracy of 98.04% and 98.00%, respectively, and an average F1-score of 98.22% and 98.20%, respectively. Given the cost and accessibility of chest X-rays, the results achieved demonstrate the potential of the proposed approach for a relatively inexpensive and accessible diagnostic method for detecting COVID-19-positive individuals. Furthermore, the use of a dataset with images collected from various sources indicates that the reported results are not constrained to a specific imaging device, but can be generalised, as also demonstrated by the very high accuracy (96.30%) achieved when classifying the images of an unseen dataset. Nevertheless, a larger study, including numerous COVID-19-positive chest X-rays, acquired from multiple radiography devices, would be required to evaluate the suitability of the proposed approach in real clinical practice.

To this end, future work will include a replication study using a much larger dataset of chest X-ray images, as well as a thorough study of the generalisation ability of the developed models by training and testing the models on diverse datasets from different sources and with X-ray images acquired by different X-ray machines. In addition, future work will also include a study on the explainability of the developed models, as well as on the use of the latest advances in deep-learning-based image classification, such as vision transformers.

## Figures and Tables

**Table 1:** Classification performance (%) of the examined deep neural network architectures following the baseline approach.

Base Model	Accuracy	F1-Score	Precision	Recall	Jaccard	Dice
EfficientNetB4	87.50	86.32	93.57	81.48	75.93	86.32
EfficientNetB7	83.75	76.39	91.45	70.86	69.40	81.94
VGG16	**92.22**	**91.94**	**93.97**	**90.13**	**83.21**	**90.84**
Xception	86.02	85.26	92.63	80.18	72.59	84.12
InceptionResNetV2	86.51	86.53	91.34	82.79	73.06	84.43
InceptionV3	86.82	84.79	90.73	80.58	72.60	84.12
MobileNetV2	86.68	85.76	91.87	81.33	72.76	84.24
ResNet50V2	89.02	88.90	93.56	85.14	77.11	87.08
DenseNet121	84.27	80.20	93.77	73.65	70.97	83.02
DenseNet169	87.23	86.52	92.84	82.03	74.75	85.55
DenseNet201	86.37	87.13	93.04	82.83	72.75	84.23

Note: Results in bold denote the best performance for each metric.

**Table 2:** Classification performance (%) of the examined deep neural network architectures following Approach 1 when using the pretrained weights for the base network and when training each network end-to-end.

Base Model	End-to-End Training						Pre-Trained Base					
	Accuracy	F1-Score	Precision	Recall	Jaccard	Dice	Accuracy	F1-Score	Precision	Recall	Jaccard	Dice
EfficientNetB4	**98.04**	**98.22**	98.52	**97.95**	**96.52**	**98.23**	89.95	88.71	92.86	85.55	80.35	89.10
EfficientNetB7	96.87	96.66	97.10	96.27	93.24	96.50	88.67	85.70	93.11	81.46	78.35	87.86
VGG16	94.77	95.39	96.11	94.84	88.83	94.08	87.57	85.59	89.23	82.86	72.85	84.29
Xception	98.00	98.20	**98.58**	97.87	95.98	97.95	90.36	90.07	92.29	88.16	79.92	88.84
InceptionResNetV2	97.38	97.35	97.88	96.89	94.17	97.00	89.05	88.76	91.25	86.65	77.51	87.33
InceptionV3	96.18	95.76	96.73	95.07	90.63	95.09	88.98	88.30	91.13	85.90	76.03	86.39
MobileNetV2	96.90	97.00	97.52	96.53	93.09	96.42	89.40	88.44	92.11	85.57	78.66	88.06
ResNet50V2	97.45	97.92	98.20	97.67	94.94	97.41	**91.02**	**90.81**	**93.43**	**88.68**	**81.23**	**89.64**
DenseNet121	97.07	97.46	97.95	97.03	93.83	96.82	88.06	87.28	93.23	83.02	75.66	86.14
DenseNet169	97.49	97.68	97.76	97.63	94.82	97.34	89.71	88.72	92.60	85.64	79.10	88.33
DenseNet201	97.25	97.15	97.60	96.77	93.93	96.87	89.12	88.25	91.63	85.58	78.21	87.77

Note: Results in bold denote the best performance for each metric and approach. Underlined results denote the overall best performance for each metric.

**Table 3:** Classification performance (%) of the examined deep neural network architectures following Approach 2 when using the pretrained weights for the base network and when training each network end-to-end.

Base Model	End-to-End Training						Pre-Trained Base					
	Accuracy	F1-Score	Precision	Recall	Jaccard	Dice	Accuracy	F1-Score	Precision	Recall	Jaccard	Dice
EfficientNetB4	96.45	96.60	97.38	95.86	92.45	96.08	93.22	92.12	95.46	89.61	86.13	92.55
EfficientNetB7	95.39	95.61	96.29	94.98	89.83	94.64	90.12	89.22	94.21	85.52	80.34	89.10
VGG16	94.66	94.83	95.61	94.15	88.70	94.01	93.94	93.74	94.40	93.13	86.28	92.64
Xception	96.72	97.26	97.80	**96.80**	93.31	96.54	93.49	93.90	94.62	93.29	86.39	92.70
InceptionResNetV2	96.70	96.12	95.83	96.58	91.68	95.66	93.94	94.02	95.63	92.57	86.23	92.60
InceptionV3	97.07	**97.27**	**98.14**	96.49	**94.59**	**97.22**	**96.18**	95.76	96.73	95.07	87.96	93.60
MobileNetV2	95.31	95.68	97.16	94.36	90.10	94.79	94.80	95.24	96.02	94.60	88.83	94.08
ResNet50V2	96.08	96.21	97.02	95.52	91.82	95.74	94.25	94.35	94.65	94.20	87.74	93.47
DenseNet121	96.18	96.55	96.88	96.25	92.89	96.31	95.42	96.06	**96.80**	95.44	90.64	95.09
DenseNet169	**97.11**	96.96	97.29	96.67	93.80	96.80	95.77	**96.12**	96.66	**95.64**	**91.17**	**95.38**
DenseNet201	96.52	96.73	97.05	96.51	92.64	96.18	95.04	95.02	95.86	94.26	89.15	94.26

Note: Results in bold denote the best performance for each metric and approach. Underlined results denote the overall best performance for each metric.

**Table 4:** Confusion matrix for the EfficientNetB4-based Approach 1 (end-to-end) without additional training or fine-tuning on the unseen COVID-19 dataset.

		Predicted		
		COVID-19	Normal	Viral Pneumonia
Actual	COVID-19	234	7	2
		(96.30%)	(2.88%)	(0.82%)
	Normal	0	0	0
		(0%)	(0%)	(0%)
	Viral pneumonia	0	0	0
		(0%)	(0%)	(0%)

**Table 5:** Execution time and size of the best-performing models.

Base Model	Approach	Trainable Parameters	Epochs	Training Time
				(s/epoch)
VGG16	Baseline	186,667,011	28	108 s
EfficientNetB4	Approach 1 (End-to-end)	17,786,571	32	112 s
Xception	Approach 1 (End-to-end)	21,077,675	20	111 s
ResNet50V2	Approach 1 (Pretrained base)	270,723	13	119 s
InceptionV3	Approach 2 (End-to-end)	28,076,195	18	123 s
Xception	Approach 2 (End-to-end)	27,114,795	21	118 s
DenseNet169	Approach 2 (Pretrained base)	5,520,643	18	166 s

Note: Average execution times measured using TensorFlow and the Keras API on the Google Colab Pro platform (Nvidia Tesla T4 and P100 GPU, 24 GB RAM). All base models were initialised with weights pretrained on the ImageNet dataset.

**Table 6:** Classification performance (%) of the best configurations versus other works using the same dataset for the same task.

Method	Accuracy	F1-Score	Precision	Recall
Aslan et al. [81] CNN	98.14	98.20	98.16	98.26
Aslan et al. [81] CNN+BiLSTM	98.70	98.76	98.77	98.76
Chowdhury et al. [54] (NIA)	97.74	96.61	96.61	96.61
Chowdhury et al. [54] (IA)	97.94	97.94	97.95	97.94
Maiti et al. [82] (GHE+TBH)	96.00	96.67	97.00	95.67
Öksüz et al. [83]	98.30	97.61	97.43	97.78
Progga et al. [84]	n/a	98.00	98.00	98.00
Sakib et al. [85]	96.00	97.67	98.00	97.67
This work-Approach 1	98.04	98.22	98.52	97.95
This work-Approach 2	97.07	97.27	98.14	96.49

NIA: no image augmentation, IA: image augmentation, GHE: global histogram equalisation, TBH: top bottom hat transform.

## Data Availability

All datasets used in this work are publicly available from their respective sources.

## Abbreviations

The following abbreviations are used in this manuscript:

BiLSTM	Bidirectional long short-term memory
CNN	Convolutional neural network
COVID-19	Coronavirus Disease 2019
CT	Computed tomography
CXR	Chest X-ray
GHE	Global histogram equalisation
GPU	Graphics processing unit
Grad-CAM	Gradient-weighted class activation mapping
IA	Image augmentation
MERS-CoV	Middle East Respiratory Syndrome
NIA	No image augmentation
PNG	Portable network graphics
RAM	Random-access memory
ReLU	Rectified linear unit
RNA	Ribonucleic acid
RT-PCR	Reverse transcription polymerase chain reaction
SARS-CoV	Severe Acute Respiratory Syndrome
SARS-CoV-2	Severe Acute Respiratory Syndrome Coronavirus 2
TBH	Top bottom hat transform
WHO	World Health Organisation
